# AXL Is a Novel Predictive Factor and Therapeutic Target for Radioactive Iodine Refractory Thyroid Cancer

**DOI:** 10.3390/cancers11060785

**Published:** 2019-06-07

**Authors:** Francesca Collina, Lucia La Sala, Federica Liotti, Nella Prevete, Elvira La Mantia, Maria Grazia Chiofalo, Gabriella Aquino, Laura Arenare, Monica Cantile, Giuseppina Liguori, Francesca Di Gennaro, Luciano Pezzullo, Nunzia Simona Losito, Giancarlo Vecchio, Gerardo Botti, Rosa Marina Melillo, Renato Franco

**Affiliations:** 1 Unità di Anatomia Patologica, Istituto Nazionale Tumori, IRCCS Fondazione G. Pascale, Via Mariano Semmola, 80131 Naples, Italy; f.collina@istitutotumori.na.it (F.C.); gabryaquino@gmail.com (G.A.); m.cantile@istitutotumori.na.it (M.C.); pinalig64@libero.it (G.L.); n.losito@istitutotumori.na.it (N.S.L.); 2 Unità di Anatomia Patologica, Università della Campania “L. Vanvitelli”, Via Luciano Armanni, 80138 Naples, Italy; lucia.ls@hotmail.it (L.L.S.); doctorelvy.lm@gmail.com (E.L.M.); renfr@yahoo.com (R.F.); 3 Dipartimento di Medicina Molecolare e Biotecnologie Mediche, Università di Napoli Federico II, Via Pansini 5, 80131 Naples, Italy; fedeliotti@hotmail.com (F.L.); vecchio@unina.it (G.V.); 4 Dipartimento di Scienze Mediche Traslazionali, Università di Napoli Federico II, Via Pansini 5, 80131 Naples, Italy; nella.prevete@unina.it; 5 Istituto per l’Endocrinologia e l’Oncologia Sperimentale “G. Salvatore”, CNR, Via Pansini 5, 80131 Naples, Italy; 6 Unità di Chirurgia della Tiroide e della Paratiroide, Istituto Nazionale Tumori, IRCCS Fondazione G. Pascale, Via Mariano Semmola, 80131 Naples, Italy; m.chiofalo@istitutotumori.na.it (M.G.C.); l.pezzullo@istitutotumori.na.it (L.P.); 7 Unità di Sperimentazioni Cliniche, Istituto Nazionale Tumori, IRCCS Fondazione G. Pascale, Via Mariano Semmola, 80131 Naples, Italy; l.arenare@istitutotumori.na.it; 8 Divisione di Medicina Nucleare, Dipartimento di Diagnostica per Immagini e Radioterapia, Istituto Nazionale Tumori, IRCCS Fondazione G. Pascale, Via Mariano Semmola, 80131 Naples, Italy; f.digennaro@istitutotumori.na.it; 9 Istituto Superiore di Oncologia, 80100 Naples, Italy; 10 Istituto Superiore di Oncologia, 16132 Genoa, Italy; 11 Direzione Scientifica, Istituto Nazionale Tumori, IRCCS Fondazione G. Pascale, Via Mariano Semmola, 80131 Naples, Italy; g.botti@istitutotumori.na.it

**Keywords:** AXL, radioactive iodine (RAI)-refractoriness, disease persistence/recurrence, p65 NF-kB activation, thyroid cancer

## Abstract

Papillary thyroid carcinomas (PTCs) have an excellent prognosis, but a fraction of them show aggressive behavior, becoming radioiodine (RAI)-resistant and/or metastatic. AXL (Anexelekto) is a tyrosine kinase receptor regulating viability, invasiveness and chemoresistance in various human cancers, including PTCs. Here, we analyze the role of AXL in PTC prognosis and as a marker of RAI refractoriness. Immunohistochemistry was used to assess AXL positivity in a cohort of human PTC samples. Normal and cancerous thyroid cell lines were used in vitro for signaling, survival and RAI uptake evaluations. 38.2% of human PTCs displayed high expression of AXL that positively correlated with RAI-refractoriness and disease persistence or recurrence, especially when combined with v-raf murine sarcoma viral oncogene homolog B(BRAF) V600E mutation. In human PTC samples, AXL expression correlated with V-akt murine thymoma viral oncogene homolog 1 (AKT1) and p65 nuclear factor kappa-light-chain-enhancer of activated B cells (NF-kB) activation levels. Consistently, AXL stimulation with its ligand growth arrest-specific gene 6 (GAS6) increased AKT1- and p65 NF-kB-phosphorylation and promoted survival of thyroid cancer cell lines in culture. Enforced expression or activation of AXL in normal rat thyroid cells significantly reduced the expression of the sodium/iodide symporter (NIS) and the radioiodine uptake. These data indicate that AXL expression levels could be used as predictor of RAI refractoriness and as a possible novel therapeutic target of RAI resistant PTCs.

## 1. Introduction

Thyroid carcinoma (TC) that derives from epithelial follicular cells represents the most common endocrine malignancy, with an increasing incidence all over the world [1]. Among thyroid malignant neoplasias, papillary thyroid carcinoma (PTCs) is the most frequent [1]. Currently, surgery followed by thyroid hormone therapy and selective use of radioactive iodine (RAI) remains the most effective therapeutic option for PTCs. However, 20–30% of PTC patients experience recurrence/persistence and/or metastasis with subsequent increased mortality [1]. Approximately 5% of metastatic PTCs lose thyroid-differentiating features, the most important of which is the expression of the sodium/iodide symporter (NIS), responsible for iodine uptake. This event allows PTCs to become radioactive iodine-refractory (RAI-R) and significantly reduces the survival rate for these tumors [1]. Several PTC molecular features have been studied to define prognostic high risk patients, including *BRAF* and rat sarcoma viral oncogene homolog (*RAS*) point mutations, *RET/PTC* and Neurotrophic tyrosine kinase, receptor (*NTRK*) rearrangements [2]. The BRAF V600E mutation, described in 28–83% of PTC specimens, has been associated to RAI refractoriness and worse outcome [1,2]. Furthermore, telomerase reverse transcriptase (*TERT*) promoter mutations have been recently described often occurring in BRAF V600E positive TC. Patients with these two combined mutations are more likely to have a poor prognosis and outcome [3].

The receptor tyrosine kinase AXL belongs to the TAM (Tyro-3, AXL, and Mer) family [4]. AXL binding to its ligand, the growth arrest-specific gene 6 (GAS6), stimulates cell proliferation and survival by triggering several signaling cascades including the mitogen-activated protein kinases (MAPK), the phosphatidylinositol 3-kinase (PI3-K)/AKT1, the Janus kinase (Jak)/ Signaling transducers and activators of transcription (Stat) and the nuclear factor kappa-light-chain-enhancer of activated B cells (NF-kB) [4] pathways. AXL was demonstrated to sustain cancer progression also independently from the expression of its ligand [4]. AXL is frequently overexpressed in human cancers [5], where it is significantly associated with a poor prognosis and therapy resistance [4]. In humans, AXL was associated with metastatic disease in lung, breast, gastric, prostate, renal cell carcinoma, and glioblastoma. Furthermore, AXL was associated to resistance to imatinib in gastrointestinal tumors, herceptin in breast cancer, and chemotherapy in acute myeloid leukemia. In addition, AXL has been demonstrated to regulate cancer angiogenesis [6]. These findings indicate that AXL displays oncogenic activities, regulating multiple key processes associated to cancer development and progression.

We previously demonstrated that AXL, often together with GAS6, is overexpressed in various TC cell lines and in a panel of 30 human PTC samples carrying different oncogenic mutations [7]. Exogenously added- or ectopically expressed-GAS6 induced AKT1, but not MAPK activation, and increased survival and proliferation of TC cell lines. Accordingly, blocking either receptor or its ligand, affected TC cell proliferation and significantly reduced their resistance to apoptotic stimuli. Consistently with our in vitro observations, silencing of AXL in TC cells significantly affected their tumorigenicity in immunocompromised mice [7]. 

With the aim to evaluate the impact of AXL on TC progression, we analyzed a series of human PTCs for AXL expression, its relation with clinico-pathologic parameters and with signal transduction pathways. We found that high expression of AXL in PTCs correlated with RAI resistance and disease persistence or recurrence. AXL tissue expression positively correlated with phospho-AKT1 and with phospho-p65 NF-kB levels. By using TC cell lines, we observed that both AKT1 and p65 NF-kB pathways are activated upon GAS6 treatment and that AXL-dependent TC cell survival in vitro depends on the activation of AKT1 and p65 NF-kB. Moreover, we found that AXL enforced expression in thyroid epithelial cells induces a decrease in Sodium Iodide Symporter NIS expression and function, indicating that AXL aberrant expression could contribute to RAI refractoriness of TC cells. Our results suggest that AXL may be used as new risk assessment tool defining PTC patients at risk of RAI refractoriness, tumor recurrence or progression. Furthermore, we defined AXL as a potential therapeutic target for RAI-R disease.

## 2. Results

### 2.1. AXL Is Expressed in Human Thyroid Cancerous Specimens, but Not in Normal Thyroid Tissues

**Figure 1:**
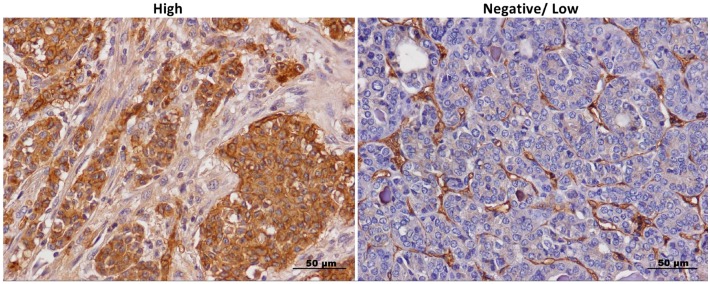
Anexeleto (AXL) staining in thyroid cancer samples. Representative images of high and low/negative AXL immunohistochemical staining in thyroid cancer samples at 40× magnification, scale bar = 50 μm. High and negative/low staining was defined as described in the Section “Materials and Methods”.

To evaluate the distribution of AXL in human PTCs and in normal thyroid tissues, we analyzed its expression in a panel of 110 PTC samples and in 5 normal thyroids by immunohistochemistry (IHC). AXL expression was considered evaluable in 102/110 PTCs Tissue MicroArray (TMA) cores. Whilst normal thyroid tissues stained negative, as also previously reported [7], the vast majority of PTCs stained AXL-positive (Figure 1 and Table 1). In all AXL positive samples, both membrane and cytosolic staining could be observed. However, some samples showed a more intense membrane than cytosolic staining, while others displayed the opposite behavior. The AXL patterns of expression were equally distributed between the two groups. PTCs exhibited high AXL staining in 38.2% (39 out of 102) of cases and low AXL expression in 61.8% (63 out of 102) of cases (Figure 1 and Table 1). Tumor stroma and non-tumor adjacent tissue staining were negative, with the exception of vascular endothelial cells, red blood cells and monocytes, as previously reported [8] (Figure 1).

**Figure 2:**
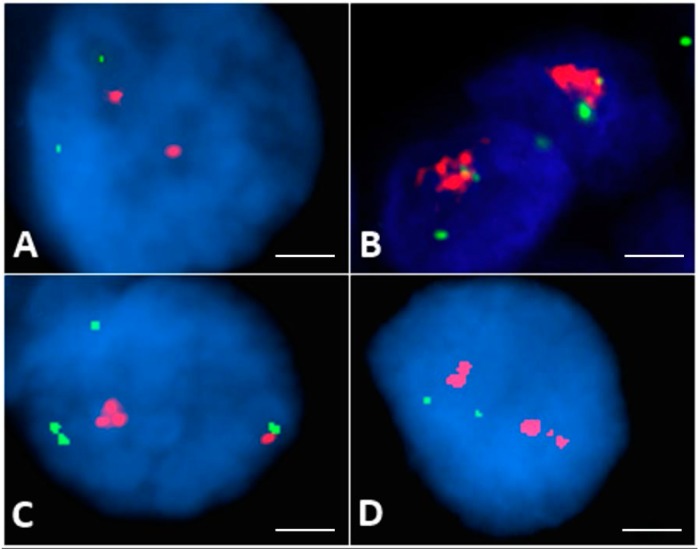
Fluorescence in Situ Hybridization (FISH) analysis of *AXL* in thyroid cancer samples. Representative FISH patterns in normal and abnormal interphase cells using the *AXL/CEN 19* probe (scale bar = 5 μm). (**A**) Normal *AXL* gene copies, two red and two green signals (2R2G); (**B**) High amplification of *AXL* gene (cluster red signals and 2G); (**C**) Low amplification of AXL gene (4R2G Ratio > 2); (**D**) Polysomic FISH patterns (3R4G).

To search for possible mechanism(s) of AXL overexpression in PTCs, we evaluated whether gene amplification could occur in *AXL* locus. Fluorescence in Situ Hybridization (FISH) analysis was evaluable in 86/110 specimens. Eighteen cases (20.9%) showed *AXL* gene amplification with *AXL/CEN19* ratio ranging from 2 to 5 (8 with high and 10 with low amplification), 4 (4.7%) exhibited polysomy, whereas 64 cases (74.4%) were normal (Figure 2). No significant correlation between AXL protein expression levels and gene amplification was observed (*p* = 0.215). In fact, within the 18 *AXL* gene-amplified specimens, high AXL protein expression was found only in three cases and low staining in the other cases; 1 out of 4 polysomic cases showed high AXL protein expression.

Thus, AXL is expressed in malignant, but not in normal, thyroid tissues. In a limited number of PTCs, AXL overexpression might be due to gene amplification. However, gene amplification does not necessarily predict AXL overexpression.

### 2.2. AXL Expression Correlates with Aggressiveness in Thyroid Carcinoma Tissues

**Figure 3:**
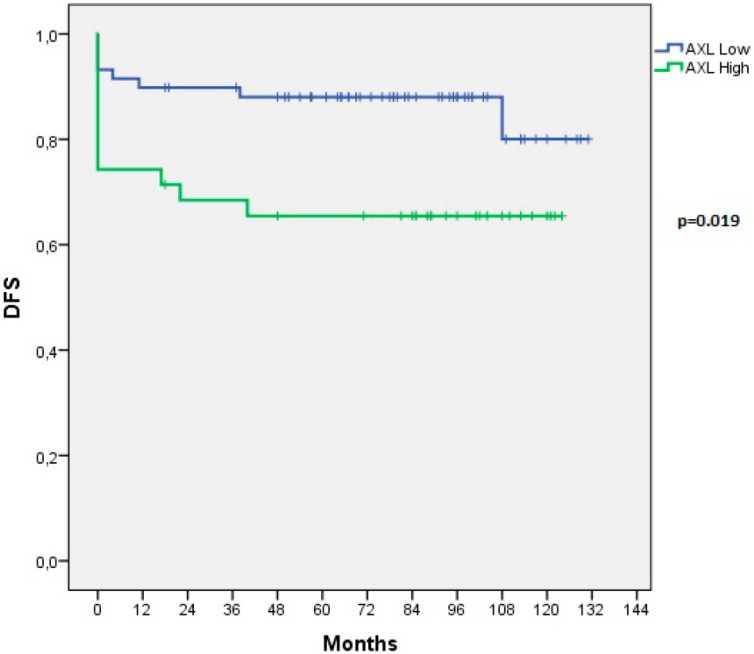
Kaplan-Meier estimation of Disease Free Survival (DFS) in thyroid cancer patients depending on AXL expression. Disease Free Survival curves of patients with papillary thyroid cancer stratified in two groups as negative/low or high AXL expression. Persisting patients were included in DFS analysis and the value 0 of the recurrence time was assigned to them in the analysis.

To evaluate the potential significance of AXL expression and its prognostic power in PTCs, we searched for the correlations between AXL expression levels and clinico-pathologic parameters. AXL expression levels did not correlate with patient age, tumor stage, size of primary tumor (T), or the presence of lymph-nodal metastases (N) (Table 1). Instead, all RAI-R tumors displayed high AXL expression (*p* < 0.0001). In order to search for association of AXL with more aggressive diseases, we looked at its expression levels in patients with complete remission compared to that with persistent or recurrent diseases. Our case pool included 14 persisting and eight recurrent diseases. High AXL expression positively correlated with disease persistence/recurrence (*p* = 0.028) (Table 1). We also evaluated the effects of AXL expression levels on Disease Free Survival (DFS) in our PTC casistic and we could demonstrate that high AXL expression inversely correlated with DFS (*p =* 0.019) (Figure 3). Similarly, an RNAseq analysis (http://www.cbioportal.org) showed that lower AXL mRNA expression was significantly associated to better overall survival and disease-free status of PTC patients [9,10].

### 2.3. Concurrent Presence of BRAF V600E Mutation and High AXL Expression Significantly Associates with RAI Refractoriness and Disease Recurrence/Persistence in Thyroid Carcinomas

Many studies used PTC-associated genetic alterations to predict tumor aggressiveness [11]. In PTCs, BRAF V600E mutation has been associated with a negative prognosis [11]. Thus, we analyzed the status of *BRAF* mutation in our PTC sample set. The BRAF V600E mutation was detected in 43.6% (48/110) PTC cases (Table 2). *BRAF* mutation status significantly associated with RAI-R tumors (*p* = 0.010, Table 2) and showed only a positive trend of statistical association with disease recurrence/persistence (*p* = 0.090), as previously shown [1]. There was no significant association with other clinico-pathologic parameters (Table 2).

As both AXL expression and BRAF mutational status were positively associated with RAI-R tumors, we asked whether the presence of BRAF V600E mutation was associated with AXL expression in our PTC samples. As shown in Table 3, BRAF V600E mutation was not significantly associated with AXL expression levels (*p* = 0.462). 

**Figure 4:**
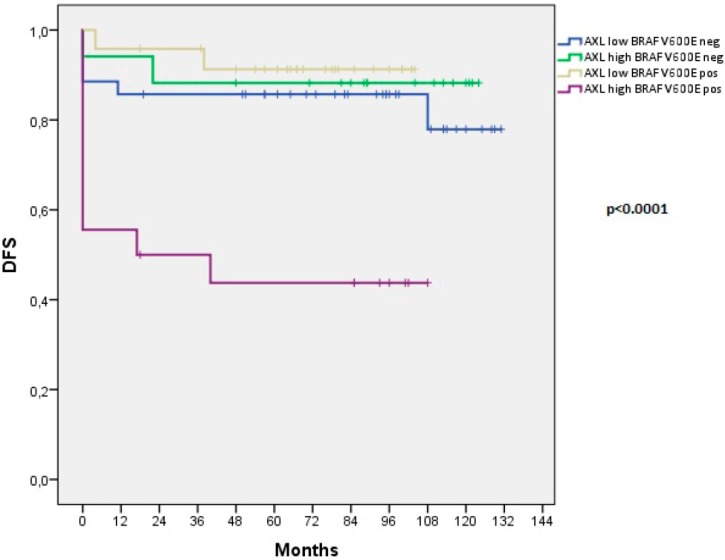
Kaplan-Meier estimation of Disease Free Survival (DFS) in thyroid cancer patients stratified for AXL expression and BRAF mutational status. Disease Free Survival curves of patients with papillary thyroid cancer stratified as: (i) AXL low + BRAF V600E negative, (ii) AXL low + BRAF V600E positive, (iii) AXL high + BRAF V600E negative, (iv) AXL high + BRAF V600E positive. Persisting patients were included in DFS analysis and the value 0 of the recurrence time was assigned to them in the analysis.

However, to understand whether the concomitant presence of BRAF V600E mutation and AXL expression could identify a subgroup of particularly aggressive PTCs, we conducted a further association analysis. We found that concomitant BRAF V600E mutation and high AXL expression correlated with RAI-R (*p* < 0.0001) and disease recurrence (*p* < 0.0001) (Table 4) more significantly than the high AXL expression or the BRAF V600E mutation singularly considered (Table 1 and Table 2). High AXL samples bearing BRAF V600E mutation showed also a significantly reduced DFS (*p* < 0.0001) compared to PTCs with only high AXL expression, BRAF V600E mutation, or with the absence of both conditions (Figure 4). 

Furthermore, to better understand the weight of AXL high expression and BRAF V600E mutation as risk factors for PTC recurrence, we conducted a multivariate analysis. The interaction between AXL and BRAF V600E on the recurrence time was significant (*p* = 0.026). The hazard ratio (HR) for patients presenting AXL high expression and BRAF V600E mutation was 4.14 with a p value of 0.007 and IC95% of 1.48–11.58; instead, it was not significant for patients who only display AXL high expression (HR = 0.59 with a *p*-value = 0.598 and IC95% of 0.13–3.2) or only BRAF V600E mutation (HR = 0.52 with a *p*-value = 0.427 and IC95% of 0.10–2.61).

Thus, we found that the concomitant presence of AXL high expression and BRAF V600E mutation could select a subgroup of patients with worse prognosis. Although AXL expression and BRAF mutational status are not statistically associated in our PTCs, we asked whether AXL expression could be dependent on BRAF activation. To this aim, we used TPC-1 and 8505c human thyroid cancer cell lines, derived from a PTC and an ATC, respectively. These cells display constitutive activation of BRAF since they carry activated RET/PTC1 and BRAF V600E oncogenes, respectively. Cells were treated or not with optimal concentrations (10 μM and 18 h) of vemurafenib, a pharmacologic BRAF inhibitor [12], for 18 h to evaluate AXL expression levels by western blot. As shown in Figure S1, BRAF inhibition by vemurafenib was able to significantly reduce AXL expression in both cell lines.

### 2.4. AXL Levels Correlate with Signaling Protein Activation 

The activation of AKT1 and ERK signaling pathways represents a central step in AXL-dependent signal transduction [13,14]. Consistently, in our previous work, we demonstrated that AXL stimulation in TC cells resulted in the activation of the AKT1 pathway, thus enhancing TC cell survival [7]. Furthermore, the GAS6/AXL/AKT1 pathway protects endothelial cells from apoptosis via the activation of NF-kB [15]. Based upon these findings, we asked whether AXL expression in TC tissues correlates with the activation of downstream signaling pathways by IHC. To this aim, we evaluated the expression levels of phosphorylated-ERK1/2, -AKT1, -p65 NF-kB that represent the active forms of the proteins. 

**Figure 5:**
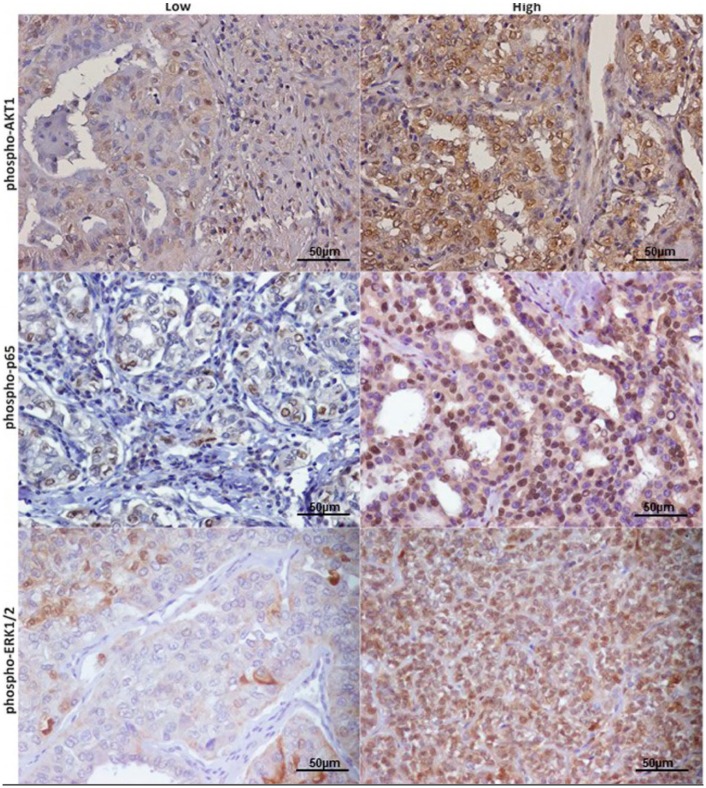
Signaling pathway activation in thyroid cancer samples. Immunohistochemical staining of representative Papillary thyroid carcinoma (PTC) samples with anti-phospho-AKT1, anti-phospho-p65 NF-kB, anti-phospho-ERK1/2 at 40× magnification, scale bar = 50 μm. High and low staining for each protein are defined as described in the Materials and Methods section.

Phospho-ERK1/2 and phospho-AKT1 staining revealed a cytoplasmic and nuclear localization (Figure 5). Phospho-p65 NF-kB showed nuclear staining (Figure 5). By analyzing these data, a significant association was observed between AXL and phospho-AKT1 (*p* = 0.030) expression levels (Table 5). In addition, AXL expression showed a positive trend of statistical association with phospho-p65 NF-kB (*p* = 0.063) levels (Table 5). We did not find any association of AXL expression with phospho-ERK1/2 levels (Table 5). 

### 2.5. AXL Activation Results in AKT1 and NF-kB p65 Phosphorylation and Protection from Apoptosis in TC Cell Lines

We previously showed that AXL is often overexpressed in TC cell lines together with its ligand GAS6, and that AXL blockade caused a significant reduction of TC cell survival. Consistently, AXL stimulation by GAS6 protected TC cells from apoptosis [7]. Here, we observed that AXL expression in TC specimens correlates with the activation of AKT1 and NF-kB p65. 

**Figure 6:**
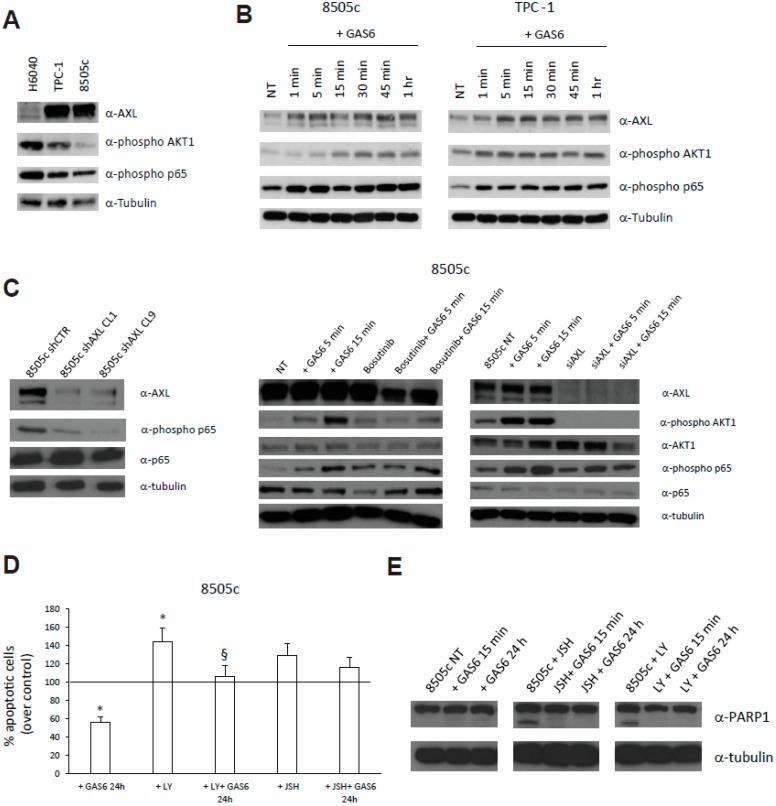
In vitro evaluation of AXL functions. (**A**) AXL, phospho-AKT1 and phospho-p65 levels evaluated in the indicated thyroid cell lines by Western Blot (WB) analysis. Anti-tubulin antibodies served as control for equal loading. (**B**) AXL, phospho-AKT1 and phospho-p65 levels in 8505c and TPC-1 cells treated with GAS6 (100 ng/mL) for the indicated time points, evaluated by WB analysis. (**C**) Levels of AXL, phospho-p65, and p65 in 8505c cell clones stably silenced for AXL (shAXL). Two clones are shown. AXL, phospho-AKT1, AKT1, phospho-p65, and p65 levels in 8505c cells treated or not with GAS6 (100 ng/mL) for the indicated time points in the presence or absence of Bosutinib (25 μM) or siRNAs targeting AXL (siAXL). Anti-tubulin antibodies served as control for equal loading. (**D**) Percent relative to control of 8505c apoptotic cells, assessed by TUNEL assay, treated or not with GAS6 (100 ng/mL) in the presence or absence of LY294002 (15 μM—AKT1 inhibitor) and JSH23 (5 μM—p65 inhibitor). * *p* < 0.05 vs. not treated cells. §, *p* < 0.05 vs. the relative control. (**E**) PARP1 cleaved levels in 8505c cells treated or not with GAS6 (100 ng/mL) in the presence or absence of LY294002 (15 μM) and JSH23 (5 μM). Anti-tubulin antibodies served as control for equal loading.

To investigate the relationship between AKT1, p65 NF-kB and AXL in TC cells, various normal and cancerous thyroid cell lines were characterized for AXL expression and activation [7]. TC cells were analyzed for their content in AXL, phospho-p65 NF-kB and phospho-AKT1. We analyzed a human primary thyroid epithelial cells (H-6040) and a normal SV40LT-immortalized thyroid cell line (N-thy). H-6040 displayed very low AXL expression levels but significant levels of AKT1 and p65 activation (Figure 6A), while N-thy expressed very high levels of AXL, phospho-Akt and phospo-p65 (Figure S2A). TPC-1 and 8505c TC cells displayed significantly higher levels of AXL but lower levels of phospho-AKT1 and p65 compared with H-6040 (Figure 6A) [7]. A wider examination for AXL, phospho-AKT1 and phospho-p65 in different TC cell lines is presented in Figure S2A. 

We have previously demonstrated that AXL stimulation in TC cell lines resulted in the activation of the AKT1 signaling pathway [7]. To establish whether AXL stimulation by its ligand GAS6 in culture also elicits p65 activation, we selected the 8505c TC cells, due to their low constitutive AXL, AKT1 and p65 activation levels, and TPC-1 cells, representative of a PTC (Figure 6B and Figure S2A). AXL stimulation by GAS6 (100 ng/mL) induced both AKT1 and p65 phosphorylation in 8505c and TPC-1 cells (Figure 6B). As a control of AXL activation upon GAS6 treatment, phospho-AXL levels, assessed by immunoprecipitation followed by western blotting with anti-phosphotyrosine antibodies, are shown in Figure S2B. Consistently, two independent AXL-depleted 8505c clones (8505c shAXL) that we previously generated [7], displayed lower constitutive phospho-p65 levels compared to control cells (Figure 6C). Moreover, acute AXL inhibition with the small molecule bosutinib (25 μM for 45 min) [16], or with specific siRNAs (24 h), reverted GAS6-induced AKT1 and p65 phosphorylation in 8505c cells (Figure 6C). Nor AXL siRNA or bosutinib, reduced the levels of phospho-p65 in unstimulated cells (Figure 6C) at the time points analyzed.

To assess the involvement of AKT1 and p65 NF-kB pathways in AXL-related cell survival, which is a well-known cellular response to AXL activation [7,14], we serum-deprived 8505c cells for 24 h to induce apoptosis, and treated them with GAS6 (100 ng/mL for 24 h) in the presence or absence of specific inhibitors of the above-mentioned pathways. As expected, GAS6 significantly reduced TC cell death as assessed by TUNEL reaction, and pre-treatment (45 min) with both LY294002 (15 μM—PI-3K/AKT1 inhibitor) [17] or JSH23 (5 μM—p65 inhibitor) [18] could revert this effect (Figure 6D and Figure S2C). Moreover, GAS6 was able to inhibit cell death signals (i.e., PARP1 cleavage) induced by both LY294002 and JSH23 (Figure 6E).

### 2.6. AXL Expression and/or Activation Status Significantly Affects NIS Expression and Function in TC Cells

Based on the significant correlation observed between AXL expression and RAI-R disease in PTCs, we hypothesized that AXL could be involved in the modulation of endogenous NIS expression/activation. The vast majority of the available TC cell lines display NIS suppression due to oncogenic signaling [19]. Thus, to study the effects of AXL on NIS expression/function, we used, as a model system, PC CL3, an immortalized clonal rat thyroid epithelial cell line, requiring TSH for growth and differentiation and expressing functional NIS, but devoid of AXL. In this cell line, we ectopically expressed human AXL by transient transfection in the presence of TSH, the major NIS stimulator, and we evaluated NIS expression by quantitative PCR and western blot. AXL transient transfection resulted in its activation assessed as its phosphorylation levels, even in the absence of ligand, due to the high levels of the receptor on the plasma membrane (Figure S3A). AXL phosphorylation levels were further increased upon GAS6 treatment (100 ng/mL for 12 h) (Figure S3A). 

**Figure 7:**
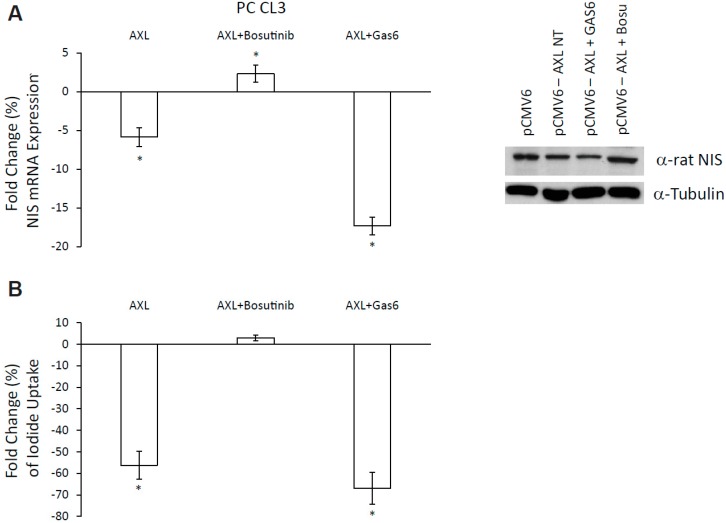
NIS modulation by AXL. (**A**) NIS mRNA expression and protein levels in PC CL3 cells transfected with pCMV6 empty vector, transfected with AXL and treated for 12 h with bosutinib (5 μM) or GAS6 (100 ng/mL). mRNA expression levels expressed as fold change (%) over control (empty vector-transfected). * *p* < 0.05 vs. the relative empty vector transfected cells. (**B**) Iodide uptake levels expressed as fold change (%) over control (empty vector-transfected) in PC CL3 cells transfected with AXL and treated with bosutinib 12 h (5 μM) or GAS6 (100 ng/mL). * *p* < 0.05 vs. the relative empty vector (pCMV6) transfected cells.

AXL enforced expression in PC CL3 cells caused a significant reduction of NIS mRNA levels compared to empty vector (pCMV6)-transfected cells. AXL-mediated NIS suppression was reverted by the addition of the AXL inhibitor bosutinib (5 μM and 12 h), indicating that AXL tyrosine kinase activity is required to inhibit NIS expression (Figure 7A). Accordingly, AXL stimulation by GAS6 (100 ng/mL for 12 h) further reduced NIS mRNA levels in AXL-transfected cells (Figure 7A) compared to the AXL transfection alone. These data were confirmed by the evaluation of endogenous NIS protein expression levels by western blot analysis, as shown in Figure 7A. Moreover, AXL enforced expression in PC CL3 caused also a significant reduction of thyroglobulin (Tg) mRNA levels compared to controls cells. GAS6 treatment (100 ng/mL—12 h) did not further decrease Tg levels. Consistently, bosutinib partially reverted the effect of AXL on Tg mRNA levels (Figure S3B).

The modulation of NIS expression may not necessarily result in significant changes in iodide uptake [20]. To assess whether not only NIS levels, but also NIS function, was affected by AXL, we evaluated the ability of PC CL3 cells to uptake iodide in culture in the presence or in the absence of the receptor. To this aim, PC CL3 cells were transiently transfected with AXL and treated with GAS6 (100 ng/mL for 12 h), bosutinib (5 μM for 12 h), or left untreated. In these conditions, we measured iodide uptake by using an established non-radioactive assay that exploits the catalytic effect of iodide on the reduction of yellow cerium to colorless cerium in the presence of arsenious acid [21]. We demonstrated that AXL ectopic expression significantly inhibited iodide uptake in PC CL3 cells and Bosutinib reverted this effect (Figure 7B). Consistently, GAS6 further decreased iodide uptake in AXL expressing PC CL3 cells (Figure 7B). Iodide uptake changes are presented in Figure 7B relative to the respective control performed on PC CL3 empty vector (pCMV6) transfected cells, arbitrarily considered as 0. These results suggest that AXL activation can inhibit NIS expression and function in TC cells thus contributing to the radioiodine resistance.

## 3. Discussion

Papillary thyroid carcinomas (PTCs) are generally characterized by an indolent growth and a good prognosis. The adjuvant treatment for PTCs is RAI ablation, which remains the best first-line therapy as long as the tumor remains iodine-avid. However, some PTC cases show a more aggressive behavior with development of lymph-nodal and visceral metastasis. Importantly, these tumors also display loss of differentiation features, including the expression/function of the iodine symporter NIS, with consequent RAI treatment failure and high mortality [1,11]. Thus, the identification of patients who need aggressive treatment and a close follow-up to reduce recurrence or persistence is necessary. Moreover, the molecular mechanisms underlying RAI refractoriness are not completely understood [2,22] and novel therapies directed at RAI resistance reversal would enrich the therapeutic tools for aggressive thyroid cancer (TC).

We found that high AXL expression levels significantly associated with RAI refractoriness, disease persistence/recurrence and reduced DFS in a cohort of human PTC samples, revealing that AXL could be a marker of TC aggressiveness. This is consistent with AXL role in other cancer histotypes, where it has been correlated with poor prognosis, increased survival and growth, invasiveness and poor response to specific anti-neoplastic treatment [5,7,14,23]. Activating genetic alterations within the AXL kinase domain are rarely found in cancer [24]. Rather, AXL expression in cancer is increased through various genetic and epigenetic mechanisms [5]. In TC, the mechanisms leading to AXL aberrant expression/activation are not completely understood. We previously demonstrated that AXL mRNA expression is positively regulated by the chemokine receptor CXCR4, a transcriptional target of the RET/BRAF/MEK/ERK pathway [25]. In a limited number of PTC samples, but not in normal thyroid tissues, we found that both AXL and its ligand GAS6 were overexpressed, suggesting that TC cells feature an AXL/GAS6 autocrine circuit [7]. In few cases, including colorectal carcinomas and sarcomas, AXL aberrant expression is due to gene amplification [23,26]. Notably, this is the first study reporting AXL gene amplification in 20.9% TC specimens and polysomy in 4.6% of samples. Our percentage is higher than that reported in TCGA data (http://www.cbioportal.org). Differently from colon cancer and sarcoma [23], in our study, gene amplification almost never correlated with AXL protein overexpression, suggesting that other transcriptional and post-transcriptional mechanisms could regulate its protein levels [27,28].

RAI refractoriness of TC is generally due to the loss of NIS [29], the iodide symporter. NIS loss of function in TC is generally caused by TC-associated oncogenic signaling pathways, including receptor tyrosine kinase aberrant activation stimulating a Receptor Tyrosine Kinase (RTK)/BRAF/MEK/ERK or PI3-K/AKT1 transduction pathway. These pathways can repress thyroid-specific transcriptional factors (TTFs), like PAX8, positive regulators of NIS transcription [30]. In fact, constitutive ERK activation is responsible for NIS repression in a fraction of TC [31], and NIS repression extent correlates with the degree of MAPK activation, which is higher in BRAF-mutant than in RTK- and RAS-mutant cancers. Accordingly, BRAF-mutant tumors are more likely to evolve into RAI-R diseases [32]. Consistently, MEK inhibitors could revert refractoriness to RAI in patients with metastatic TC [33,34]. In addition, post-transcriptional mechanisms can also impede NIS function by suppressing its glycosylation and altering its membrane trafficking [35,36].

Here, we identify AXL as a possible additional factor that contributes to RAI resistance in TC. In fact, in our set of PTC samples, AXL expression correlates with RAI-R status, as did BRAF mutational status, to date the best established risk factor for RAI-R disease. Importantly, the presence of BRAF V600E mutation together with AXL high expression significantly increased the association with RAI refractoriness and disease persistence or recurrence. Although the two markers were not statistically linked, we found that BRAF inhibition by Vemurafenib significantly reduced AXL expression in culture. Moreover, the multivariate analysis shows that AXL high expression and BRAF V600E mutation are not independent risk factors for DFS, increasing the risk of disease persistence or recurrence by almost five times.

The coexistence of BRAF mutation with TERT promoter mutations has also been associated with poor clinical outcomes in TC. Thus, it will be important to assess the status of TERT and its association with BRAF V600E and AXL expression in our sample set.

One limitation of our study is the small number of patients in our cohort. Further studies on larger samples set will be necessary to confirm our findings. However the experiments on thyroid cells in culture support our data. AXL enforced expression in cultured rat normal thyroid cells (PC CL3) repressed NIS expression and NIS iodide uptake ability, and AXL inhibition with Bosutinib reverted this effect. Both AKT1 and NF-kB signaling, as well as other signaling pathway(s) downstream AXL could be responsible for RAI resistance, and we are currently investigating this issue. 

Epigenetic modifications have been identified as being responsible for the de-differentiation of thyroid tissue and its malignant transformation. In particular, it has been shown that NIS is under epigenetic control [37]. Our preliminary data suggest that there is no involvement of methylation or deacetylation in AXL induced NIS regulation. In any case, we cannot exclude the possibility that AXL could modulate NIS expression through other epigenetic mechanisms.

AXL is overexpressed in several hematological and solid malignancies [5]. Furthermore, several evidence suggests that AXL overexpression drives wide-ranging processes: epithelial to mesenchymal transition, angiogenesis, resistance to chemotherapic and targeted agents, and decreased anti-tumor immune response [38]. Thus, AXL is already considered an attractive candidate as both a prognostic biomarker and a target for anti-cancer therapies in several cancer models. In fact, several AXL inhibitors are currently used in preclinical and clinical development [5]. Relatively to TC, the greatest promise for optimal treatment of this neoplasia remains the identification of markers useful to identify patients at risk of recurrence, metastasis and RAI resistance. This is the first demonstration that AXL could be considered both a promising prognostic marker and a therapeutic target for TC patients that feature RAI-R disease. Animal models of TC will aid in defining the effects of AXL inhibition on iodine sensitivity in vivo, and will open the possibility of translating AXL inhibitory therapies in patients with RAI-R TC.

## 4. Materials and Methods

### 4.1. Patients and Specimens 

From 2004 to 2013, 110 patients diagnosed with PTC and 5 normal thyroid tissues were selected from the Pathology Unit, National Cancer Institute “Fondazione G. Pascale” of Naples and enrolled in this study. All cases were reviewed according to WHO classification criteria [39]. Medical records were reviewed for clinico-pathologic information that are summarized in Table 6.

Thirteen patients were classified as having RAI-R disease when they had: (i) lack of RAI uptake on post-therapy scan after RAI-administered activity >30 mCi following appropriate iodine deprivation and adequate thyrotropin (TSH) elevation; (ii) lack of RAI uptake on a properly conducted diagnostic whole-body scan in the setting of know structural disease, as demonstrated by cross-sectional imaging; (iii) lack of demonstrable ability of the tumor to concentrate sufficient RAI for tumoricidal effect, based on lesional dosimetry (i.e., delivered RAI dose to metastatic foci <8000 cGy) when available; (iv) continued progression of thyroid cancer, despite cumulative RAI-administered activities >500–600 mCi in adult patients [40]. 

Recurrent patients displayed disease-free status at least 1 year after treatment. Disease-free status was defined according with the ATA guidelines as absence of clinical, imaging and biochemical evidence of disease. Persistent disease was defined as presence of positive Tg, persisting/increasing Tg antibodies or presence of structural disease within 1 year after initial treatment (surgery with or without ^131^I ablation) [41]. The mean follow-up period was 7 years. In our persisting patients, the value of Tg was between 3 and 452 ng/mL within 1 year after treatment; in recurrent patients, Tg levels ranged from 0 to 0.8 ng/mL up to 1 year after initial treatment.

Informed consent for the scientific use of biologic material was obtained from all patients and the work was approved by the local Ethical Committee of Istituto Nazionale Tumori “Fondazione G. Pascale” (CEI 556/10 of 12/3/2010).

### 4.2. BRAF V600E Mutation Analysis

The DNA isolation was performed on two 10 μm-thick paraffin tissue sections through the QIAamp DNA FFPE Tissue Kit (QIAGEN, Dusseldorf, Germany), in accordance with the protocols of the manufacturing companies. The DNA was measured using NanoDrop and found to be adequate for the analysis and preserved at −20 °C until the relevant analyses were performed. A melt curve analysis was conducted in the LightCycler^®^ (Roche, Berlin, Germany) 480 instrument using the ready kit LightMix^®^ Kit *BRAF*^V600E^ (Roche^®^, Berlin, Germany) for the *BRAF V600E* mutation analysis.

### 4.3. Tissue Microarray Building

A thyroid Tissue MicroArray (TMA) included 110 tumor tissue samples and five normal thyroid tissue as controls and was constructed using the most representative areas from each case. All tumors and controls were reviewed by two experienced pathologists (R.F. and E.L.M.). Tissue cylinders, with a diameter of 1 mm, were punched from morphologically representative tissue areas of each ‘donor’ tissue block and brought into one recipient paraffin block (3 × 2.5 cm) using a semiautomated tissue array (Galileo TMA, ISENET, Milan, Italy) [42,43].

### 4.4. Immunohistochemical Analysis

Immunohistochemical (IHC) staining was performed on slides from formalin-fixed, paraffin embedded tissues, corresponding to cases of thyroid carcinomas and normal thyroid tissues to evaluate the expression of AXL (1:20 dilution; R&D System Europe, Ltd, Abingdon, UK), phospho-AKT1 (Ser473) (1:150 dilution; Abcam, Cambridge, UK), -ERK1/2 (Thr202/Tyr204) (1:500 dilution; Cell Signaling, Leiden, The Netherlands), -p65 NF-kB (Ser536) (1:250 dilution; Abcam, Cambridge, UK). The peroxidase reactivity was visualized using a 3,3′-diaminobenzidine (DAB). Immunohistochemical expression was evaluated by four expert pathologists (Renato Franco, Elvira La Mantia, Gerardo Botti and Nunzia Simona Lo Sito) using a light microscopy and evaluating the intensity and the cells percentage of immunostaining. Observers was unaware of the clinical outcome. For each sample, at least five fields (inside the tumor and in the area exhibiting tumor invasion) and >500 cells were analyzed [44]. 

A standardized score for these markers doesn’t exist, and the median positivity for each marker was used as cut-off. Thus, our score evaluation was the following: for predominantly membranous and cytoplasmic AXL staining we considered cell percentage positivity as negative/low when it was ≤10%, high when >10%; for nuclear phospho-p65 (Ser536) as low when it was ≤50% and high when >50%; for cytoplasmic and nuclear phospho-AKT1 as low when it was <20% and high when ≥20%; for phospho-ERK1/2 positivity was considered high in presence of nuclear and cytoplasmic staining ≥30%, low when the nuclear staining was absent and cytoplasmic staining <30%.

### 4.5. FISH Analysis

Tissue array sections from paraffin-embedded tissue were heated for 4 h at 62 °C and immediately deparaffinized in two rinses of 100% xylene for 10 min each. *AXL/CEN19q* FISH Probe (FG0088, Abnova, Tapei City, Taiwan) (10 μL) was applied to the tissue sections. The *AXL/CEN19q* FISH probe is a mixture of a Texas Red-labeled DNA probe covering a ~290 kb region of AXL locus, and a fluorescein-labeled DNA probe covering a ~430kb centromeric region of chromosome 19. Slides were counterstained with 4′6-diamidino-2-phenylindole dihydrochloride (DAPI). FISH was performed according to the manufacturer’s instructions (Vysis, Abbott Molecular, Chicago, IL, USA). FISH data were collected using an Olympus BX 61 fluorescence microscope equipped with a cooled black-and-white camera controlled by the associated software (Olympus, Milan, Italy). Signals were evaluated by three independent evaluators (A.G., L.G. and F.R.) scoring at least 60 interphase nuclei in four different high power fields (HPF). The FISH results were scored as follow: specimens with the ratio *AXL/CEN19* ≥ 2.0 were considered as amplified; we considered polysomic the cases showing, in more than 30% of the evaluated cells, three or more CEN19 signals and with an *AXL/CEN19* ratio of 1 for cell.

### 4.6. Cell Cultures

The human normal immortalized thyroid cell line N-thy, the thyroid papillary cancer cell lines TPC-1 and NIM-1 and the anaplastic thyroid cancer cell lines 8505c, FB1, SW1736, and ACT-1 were obtained from Giancarlo Vecchio and cultured as described previously [42]. Human primary thyroid epithelial cells H-6040 cells were from Cell Biologics (Chicago, IL, USA) and were grown in Human Epithelial Cell Medium supplemented with TSH (2 μU/mL) and the Human Epithelial Cell Medium Supplement Kit (Cell Biologics Cat. No. H6621) containing 0.5 mL Insulin-Transferrin-Selenium (ITS), 0.5 mL EGF, 0.5 mL hydrocortisone, 5.0 mL L-glutamine, 5.0 mL Antibiotic-Antimycotic Solution, 10.0 mL Epithelial Cell Supplement, 50.0 mL FBS. For these cells thyroid identity was assessed by evaluating the levels of thyroid differentiation markers, including Tg, NIS and TSHR, by RT-PCR. PC CL3 is a differentiated thyroid epithelial cell line derived from 18-month-old Fischer rats, was kindly provided by Alfredo Fusco and maintained as described elsewhere [45]. For AXL-RNA interference, SMARTpools (a pool of four custom-synthesized siRNAs) by Dharmacon (Lafayette, CO, USA) or Mission shRNAs (a pool of five lentiviral contructs) by Sigma Aldrich (St. Louis, MO, USA) were used as previously described [7]. pCMV6 AXL plasmid was from Origene (Rockville, MD, USA). Treatment of TC cell lines with GAS6 (R&D Systems, Minneapolis, MN, USA Cat #885-G SB), Bosutinib (Selleckchem Houston, TX, USA Cat # SKI-606), LY294002 (Cell Signaling Cat #9901) or JSH-23 (Sigma Aldrich Cat #J4455) were done upon serum deprivation.

### 4.7. Protein Studies

Cells were lysates as described elsewhere [42]. Protein extractions, immunoblotting, and immunoprecipitation assays were carried out according to standard procedures [7]. Anti-AXL was from Santa Cruz Biotechnology (Dallas, TX, USA); anti-p65 and anti-phospho-p65 (Ser536), anti-AKT1 and anti-phospho-AKT1 (Ser473), anti-cleaved PARP1 were from Cell Signaling, anti-phosphotyrosine antibody (4G10) was from Merk Millipore (Darmstadt, Germany). Monoclonal anti-tubulin was from Sigma Chemical Co (Milan, Italy). Rat NIS levels were studied by using anti-rat NIS antibody from Sigma Aldrich. Secondary anti-mouse, anti-goat, and anti-rabbit antibodies coupled to horseradish peroxidase were from Bio-Rad (Munich, Germany).

### 4.8. TUNEL Assay

For TUNEL, an equal number (5 × 10^3^) of cells was seeded onto single-well Costar glass slides; cells were serum-deprived for 24 hours and then treated for 24 hours with GAS6 (100 ng/mL) in the presence or absence of LY294002 (15 μM) [17] and JSH23 (5 μM) [18]. Finally, cells were subjected to the TUNEL reaction (Roche^®^, Berlin, Germany) as described elsewhere [46].

### 4.9. RNA, cDNA and Real-Time-PCR

Total RNA was isolated and retro-transcribed as previously described [47]. Quantitative PCR was performed by using the SYBR Green PCR Master mix (Applied Biosystems, Foster City, CA, USA) in the iCycler apparatus (Bio-Rad, Hercules, CA, USA). Amplification reactions (25 μL final reaction volume) contained 200 nM of each primer, 3 mM MgCl_2_, 300 μM dNTPs, 1× SYBR 37 Green PCR buffer, 0.1 U/μL AmpliTaq Gold DNA Polymerase (Applied Biosystems), 0.01U/μL Amp Erase (Applied Biosystems), RNase-free water and 100 ng cDNA samples. Thermal cycling conditions were optimized for each primers pair [47]. Normalization was performed using β-actin mRNAs levels. The rat NIS primers were: forward 5′-GCTGTGGCATTGTCATGTTC-3′, and reverse 5′-TGAGGTCTTCCACAGTCACA-3′, the rat TG primers were: forward 5′-GAGTGATGCTCCC AGCTTCT-3′, and reverse 5′-AGTTCCTGGTGGCTGAAATG-3′, the rat β-actin primers were: forward 5′-GTCAGGCAGCTCATAGCTCT-3′, and reverse 5′-TCGTGCCGTGACATTAAAGAG-3′.

### 4.10. Iodide Uptake Assay

Iodide uptake and efflux study was performed as described previously [21] with minor modifications. After 48 h of AXL transfection in combination or not with GAS6 (100 ng/mL and 12 h) or bosutinib (5 μM and 12 h) [16] treatment, cells were incubated with 10 μM NaI in uptake buffer (Hank’s Balanced Salt Solution (HBSS) supplemented with 10 mM HEPES (pH 7.3)). After 30 min incubation with NaI at 37 °C, cells were washed with ice-cold uptake buffer. Then 10.5 mM ammonium cerium (IV) sulphate solution and 24 mM sodium arsenite (III) solution were added. The plate was incubated at room temperature (RT) in dark for 30 minutes and the absorbance at 420 nm was recorded. Using logarithmic conversion and standard equation of iodide standards, amount of iodide uptake was calculated and expressed as fold change (%) over control.

### 4.11. Statistical Analysis

The Pearson χ^2^ test was used to determine whether a relationship exists between the categorical variables included in the study and AXL level expression and BRAF V600E mutation. All statistical tests were two tailed and the level of significance was defined as *p* < 0.05. Disease-free survival (DFS) curve was plotted using the Kaplan-Meier method with significance valuated using a stratified Mantel-Cox log-rank test. DFS was measured as the time from the surgery until the date of the progression occurrence, relapse after complete remission, or death from any cause. In DFS analysis, recurrence times had a value of 0 for patients who did not achieve complete remission. 

Stratified Multivariable Cox proportional hazards models were used to estimate hazard ratios (HR) and 95% confidence intervals (95% CI), including AXL expression, BRAF mutational status and interaction between AXL and BRAF. Finally, to allow interpretation of interaction, we showed the HR for the different categories of AXL expression and/or BRAF mutational status. 

Bivariate statistical analyses and survival analysis were carried out using the Statistical Package for Social Science v. 20 software (SPSS Inc., Chicago, IL, USA), while the multivariate analyses were performed using Stata/MP for Windows (version 14.2).

## 5. Conclusions

Here, we provide data indicating that: (i) the AXL receptor tyrosine kinase may represent a novel prognostic tool defining high-risk PTC patients, especially when combined with the presence of the BRAF V600E mutation; (ii) AXL receptor tyrosine kinase may represent a novel potential therapeutic target, as its blocking is capable of inhibiting TC cell survival and restore NIS function, thus favoring the recovery of TC RAI sensitivity.

## Figures and Tables

**Table 1:** Relation between AXL expression and clinico-pathologic features in the group of 102 patients. RAI-R = Radioactive Iodine-Refractoriness. * *p* ≤ 0.05; *** *p* ≤ 0.001.

Number of Patients with PTC	AXL (*n* = 102)		*p*-Value
	Low (*n* = 63)	High (*n* = 39)	
Age			
<45	36 (60%)	24 (40%)	0.661
≥45	27 (64.3%)	15 (35.7%)	
Stage			
I–I	45 (61.6%)	28 (38.4%)	0.968
III–V	18 (62.1%)	11 (37.9%)	
Tumor size			
T1–2	31 (62%)	19 (38%)	0.962
T3–4	32 (61.5%)	20 (38.5%)	
Nodal status			
N0	33 (61.1%)	21 (38.9%)	0.885
N1	30 (62.5%)	18 (37.5%)	
RAI-R			
No	63 (70%)	27 (30%)	<0.0001 ***
Yes	0 (0%)	12 (100%)	
Disease Persistence or Recurrence			
No	54 (66.7%)	27 (35.3%)	0.028 *
Yes	8 (40%)	12 (60%)	
N/A	1 (100%)	0 (0%)	

**Table 2:** Relation between v-raf murine sarcoma viral oncogene homolog B (BRAF) mutation status and clinico-pathologic features in the group of 110 patients. RAI-R = Radioactive Iodine-Refractoriness. ** *p* ≤ 0.01.

Number of Patients with PTC	BRAF V600E Mutation (*n* = 110)		*p*-Value
	No (*n* = 62)	Yes (*n* = 48)	
Age			
<45	35 (54.7%)	29 (45.3%)	0.676
≥45	27 (58.7%)	19 (41.3%)	
Stage			
I–II	43 (55.1%)	35 (64.9%)	0.683
III–IV	19 (59.3%)	13 (40.7%)	
Tumor size			
T1–T2	33 (60%)	22 (40%)	0.442
T3–T4	29 (52.7%)	26 (47.3%)	
Nodal status			
N0	34 (55.7%)	27 (44.3%)	0.883
N1	28 (57.1%)	21 (42.9%)	
RAI-R			
No	59 (60.8%)	38 (39.2%)	0.010 **
Yes	3 (23.1%)	10 (76.9%)	
Disease Persistence or Recurrence			
No	53 (60.9%)	34 (39.1%)	0.090
Yes	9 (42.9%)	13 (57.1%)	
N/A	0 (0%)	1 (100%)	

**Table 3:** Relation between AXL expression and BRAF V600E mutational status.

Mutational Status	AXL (*n* = 102)		*p*-Value
	Low (*n* = 63)	High (*n* = 39)	
BRAF V600E mutation			
Negative	37 (64.9%)	20 (35.1%)	0.462
Positive	26 (57.8%)	19 (42.2%)	

**Table 4:** Correlation analysis with clinico-pathologic features in the group of patients expressing both BRAF V600E mutation and high AXL expression. RAI-R: Radioactive Iodine-Refractoriness. *** *p* ≤ 0.001.

Number of Patients with PTC	High AXL Expression and BRAF V600E Mutation (*n* = 102)		*p*-Value
	No (*n* = 83)	Yes (*n* = 19)	
Age			
<45	48 (80%)	12 (20%)	0.670
≥45	35 (83.3%)	7 (16.7%)	
Stage			
I–II	58 (79.5%)	15 (20.5%)	0.429
III–IV	25 (86.2%)	4 (13.8%)	
Tumor size			
T1–T2	43 (86%)	7 (14%)	0.239
T3–T4	40 (76.9%)	12 (23.1%)	
Nodal status			
N0	46 (85.2%)	8 (14.8%)	0.294
N1	37 (77.1%)	11 (22.9%)	
RAI-R			
No	81 (90%)	9 (10%)	<0.0001 ***
Yes	2 (16.7%)	10 (83.3%)	
Disease Persistence or Recurrence			
No	72 (88.9%)	9 (11.1%)	<0.0001 ***
Yes	10 (50%)	10 (50%)	
N/A	1 (100%)	0 (0%)	

**Table 5:** Relation between AXL marker and phospho-ERK 1/2, phospho-AKT1, phospho-p65 NF-kB expression in thyroid cancer patients. * *p* < 0.05.

Signaling Pathways	AXL (*n* = 102)		*p*-Value
	Low (*n* = 63)	High (*n* = 39)	
phospho-ERK1/2			
Negative	10 (45.5%)	12 (54.7%)	0.118
Positive	40 (64.5%)	22 (35.5%)	
N/A	13 (72.2%)	5 (27.8%)	
phospho-AKT1			
Low	48 (66.7%)	24 (33.3%)	0.030 *
High	9 (40.9%)	13 (59.1%)	
N/A	6 (75%)	2 (25%)	
phospho-p65 NF-kB			
Low	40 (71.4%)	16 (28.6%)	0.063
High	20 (52.6%)	18 (47.4%)	
N/A	3 (37.5%)	5 (62.5%)	

**Table 6:** Main clinico-pathologic data of PTC patients.

Number of Patients with PTC	110
Age	
<45	64 (58.2%)
≥45	46 (41.8%)
Sex	
Male	26 (23.6%)
Female	84 (76.4%)
Histologic Variants	
Classic type	90 (81.8%)
Follicular variant	20 (18.2%)
Tumor size	
T1	31 (28.2%)
T2	24 (21.8%)
T3	53(48.2%)
T4	2 (1.8%)
Nodal status	
N0	61 (55.5%)
N1	49 (44.5%)
Stage	
I	73 (66.4%)
II	5 (4.5%)
III	23 (20.9%)
IV	9 (8.2%)
Disease Recurrence or Progression	
No	87 (79.1%)
Yes	22 (20%)
Unknown	1 (0.9%)

## Supplementary Materials

The following are available online at https://www.mdpi.com/2072-6694/11/6/785/s1, Figure S1: AXL levels evaluated in the 8505c and TPC-1 cells treated or not with Vemurafenib (Vemu-10 μM-18 h) by WB analysis, Figure S2: (A) AXL, phospho-AKT1 and phospho-p65 levels evaluated in the indicated thyroid cell lines by WB analysis. Anti-tubulin antibodies served as control for equal loading. (B) Analysis of AXL levels and tyrosine-phosphorylation by AXL-immunoprecipitation followed by WB with anti-phosphotyrosine (phospho-Tyr) and anti-AXL in 8505c and TPC-1 cells left untreated or treated with GAS6 (100 ng/mL) for the indicated time points. (C) Representative photograms of 8505c apoptotic cells, assessed by TUNEL assay, untreated (NT) treated with GAS6 (100 ng/mL) in the presence or absence of LY294002 (15 μM—AKT1 inhibitor) and JSH23 (5 μM—p65 inhibitor), Figure S3: (A) Analysis of AXL levels and tyrosine-phosphorylation by AXL-immunoprecipitation followed by WB with anti-AXL and anti-phosphotyrosine (phospho-Tyr) in PC CL3 cells transfected with pCMV6 empty vector, transfected with AXL and treated for 12 hours with Bosutinib (5 μM) or GAS6 (100 ng/mL). (B) Thyroglobulin mRNA levels inhibition expressed as (%) over control (empty vector—transfected) in PC CL3 cells transfected with AXL and treated for 12 hours with Bosutinib (5 μM) or GAS6 (100 ng/mL).
