# Supplementary material for: AXL Is a Novel Predictive Factor and Therapeutic Target for Radioactive Iodine Refractory Thyroid Cancer
Source: Cancers (Basel). 2019 Jun 7;11(6):785. doi: 10.3390/cancers11060785 (PMC6628138; doi:10.3390/cancers11060785)
Supplement: Supplementary file 1 [file cancers-11-00785-s001.pdf]

Supplementary Materials: AXL Is a Novel Predictive Factor and Therapeutic Target for Radioactive Iodine Refractory Thyroid Cancer

Francesca Collina, Lucia La Sala, Federica Liotti, Nella Prevete, Elvira La Mantia, Maria Grazia Chiofalo, Gabriella Aquino, Laura Arenare, Monica Cantile, Giuseppina Liguori, Francesca Di Gennaro, Luciano Pezzullo, Nunzia Simona Losito, Giancarlo Vecchio, Gerardo Botti, Rosa Marina Melillo and Renato Franco

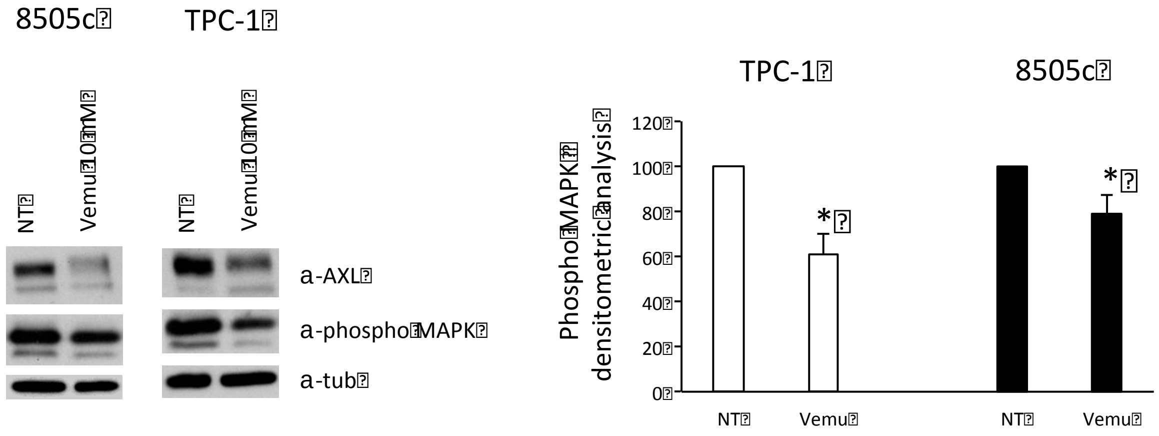

Figure S1. AXL levels evaluated in the 8505c and TPC-1 cells treated or not with Vemurafenib (Vemu -10 µM-18 h) by WB analysis. Anti-tubulin antibodies served as control for equal loading, anti-phospho-MAPK levels as control of Vemurafenib effects. The levels of phospho MAPK were also evaluated by densitomeric analysis of three independent experiments. * $p < 0.05$ compared to untreated (NT) cells.

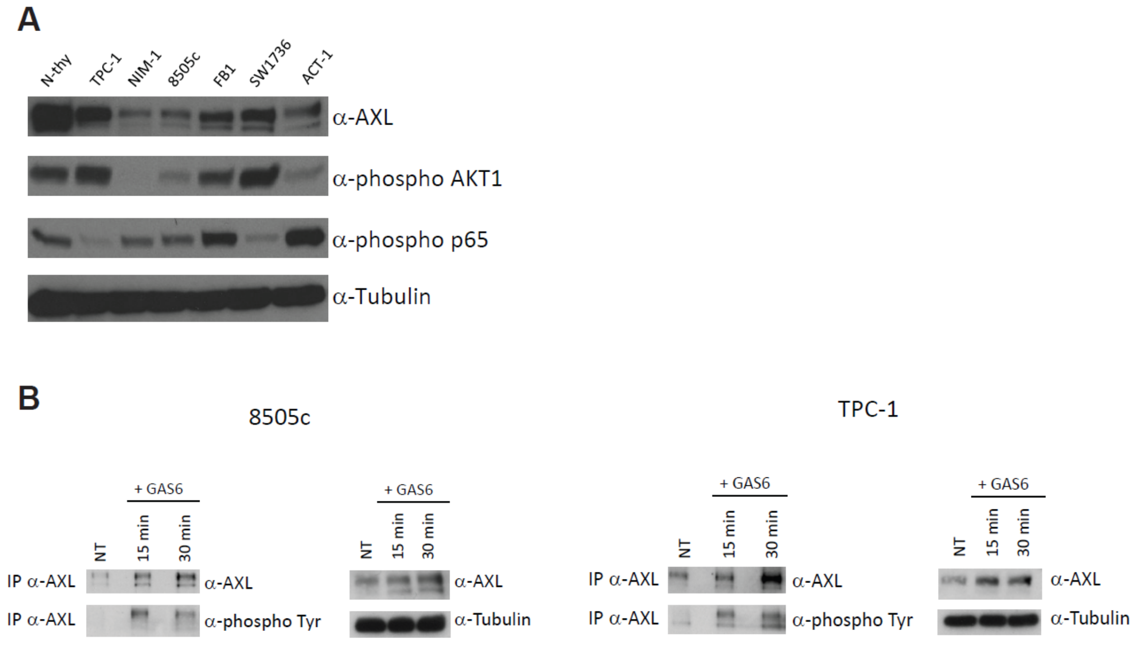

Figure S2. *Cont.*

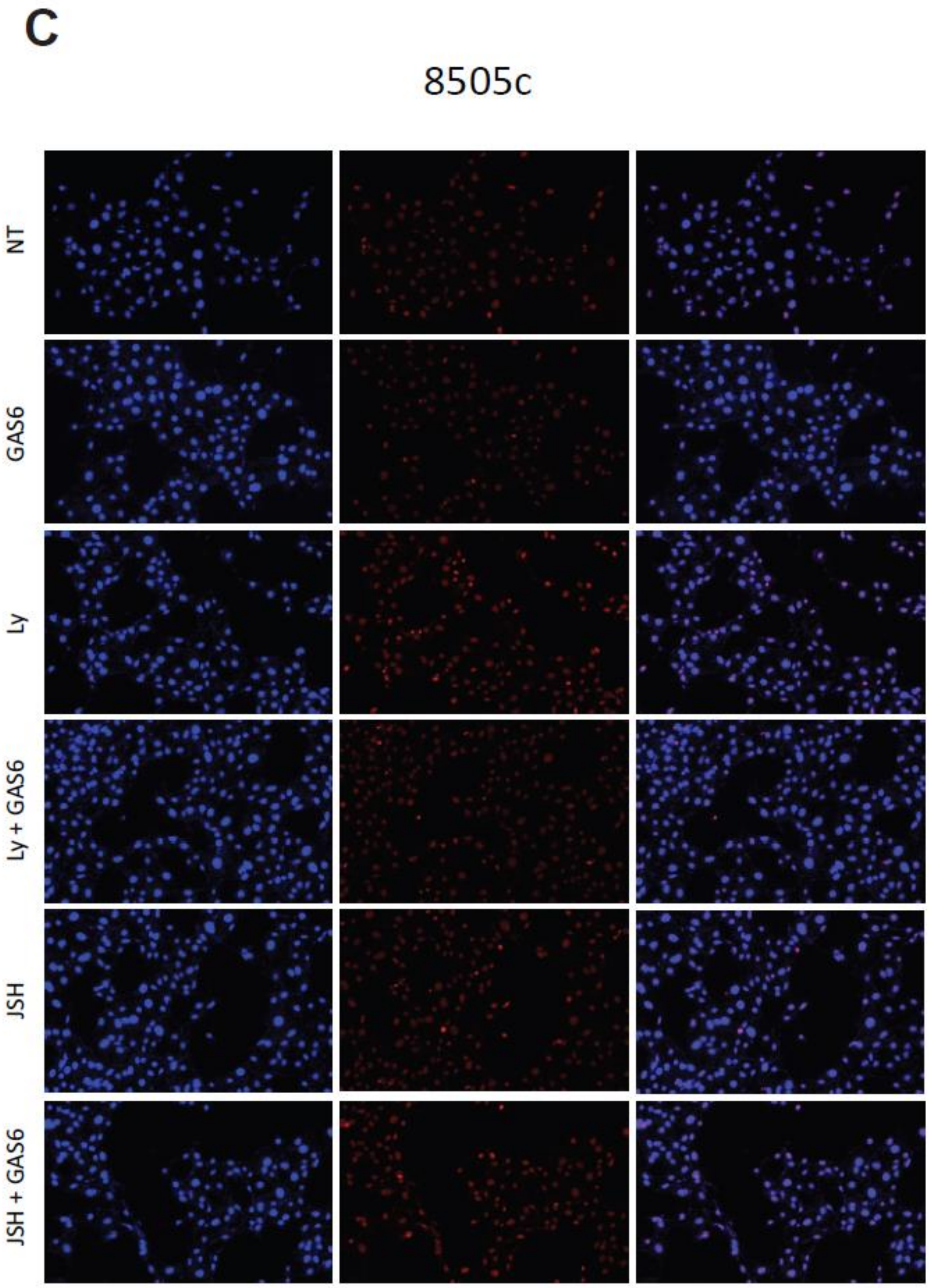

Figure S2. (**A**) AXL, phospho-AKT1 and phospho-p65 levels evaluated in the indicated thyroid cell lines by WB analysis. Anti-tubulin antibodies served as control for equal loading. (**B**) Analysis of AXL levels and tyrosine-phosphorylation by AXL-immunoprecipitation followed by WB with anti-phosphotyrosine (phospho-Tyr) and anti-AXL in 8505c and TPC-1 cells left untreated or treated with GAS6 (100 ng/mL) for the indicated time points. (**C**) Representative photograms of 8505c apoptotic cells, assessed by TUNEL assay, untreated (NT) treated with GAS6 (100 ng/mL) in the presence or absence of LY294002 (15 µM—AKT1 inhibitor) and JSH23 (5 µM—p65 inhibitor). Scale Bar: 50 µM.

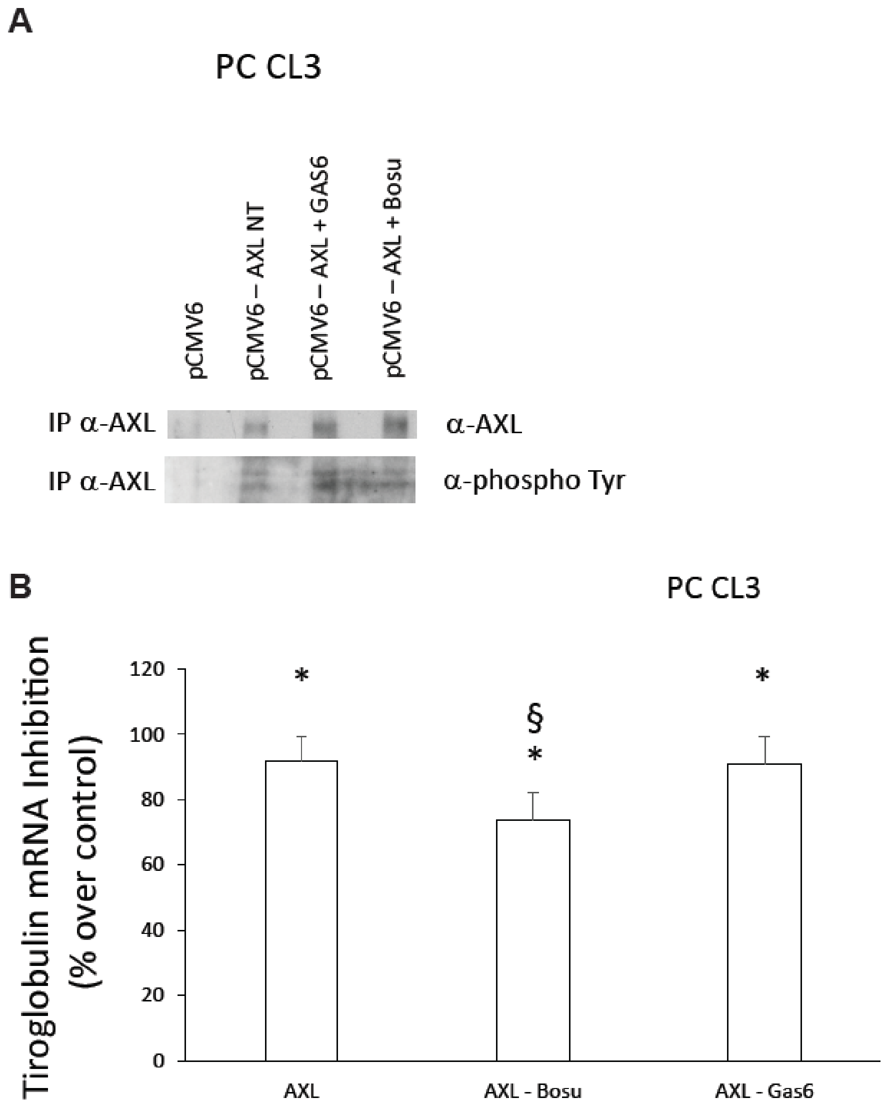

Figure S3. (**A**) Analysis of AXL levels and tyrosine-phosphorylation by AXL-immunoprecipitation followed by WB with anti-AXL and anti-phosphotyrosine (phospho-Tyr) in PC CL3 cells transfected with pCMV6 empty vector, transfected with AXL and treated for 12 hours with Bosutinib (5 μM) or GAS6 (100 ng/mL). (**B**) Thyroglobulin mRNA levels inhibition expressed as (%) over control (empty vector—transfected) in PC CL3 cells transfected with AXL and treated for 12 hours with Bosutinib (5 μM) or GAS6 (100 ng/mL). * $p < 0.05$ vs. the relative empty vector (pCMV6) transfected cells. §: $p < 0.05$ vs AXL alone
